# Exposure to Vape Products Elicits Neural Activity Patterns Indicative of Approach Motivation Among Young People

**DOI:** 10.1111/adb.70130

**Published:** 2026-02-16

**Authors:** Stefan Bode, Daniel Feuerriegel, Jane Yook, Michelle I. Jongenelis

**Affiliations:** ^1^ Psychology, Division of Science New York University Abu Dhabi Abu Dhabi UAE; ^2^ Melbourne School of Psychological Sciences The University of Melbourne Parkville Victoria Australia; ^3^ Melbourne Centre for Behaviour Change, Melbourne School of Psychological Sciences The University of Melbourne Parkville Victoria Australia

**Keywords:** appeal, e‐cigarettes, electroencephalography, neural decoding, vapes

## Abstract

Previous research exploring the impact of exposure to e‐cigarette (vape) products has been limited by a reliance on self‐report measures, responses to which require self‐reflection and insight. The present study sought to address these limitations by using novel ‘neural decoding’ techniques to establish whether exposure to e‐cigarette products triggers automatic and unconscious product approach tendencies. An electroencephalography (EEG) study was conducted in Australia. Participants (*n* = 38; 68% women; 17–23 years old) were presented with images of e‐cigarette products for several seconds while their brain activity was recorded. The primary outcome variables were brain activity and ratings of product appeal, curiosity and wanting. Results suggest that young people's vape approach tendencies can be predicted from brain activity as early as 100–300 ms after exposure, and then again over a sustained time period 350–800 ms after exposure. These findings were not restricted to those who currently vape, with similar patterns observed in young people who had never vaped or had tried vaping products in the past. Findings provide neuroscientific evidence for the presence of fast and automatic processing of vape products, which results in immediate approach tendencies. This suggests that approach attitudes and behavioural intentions that occur in response to exposure to vape products precede deliberate processing, and that exposure to e‐cigarette products has an immediate effect on young people's neural processing. This novel neural approach to understanding marketing for e‐cigarettes offers valuable insights into how fast and automatic product‐based marketing of e‐cigarettes unfolds in young people's brains, with implications for product regulation.

## Graphical abstract

We used electroencephalography and novel ‘neural decoding’ techniques to establish whether exposure to e‐cigarette products triggers automatic and unconscious product approach tendencies. Results show that young people’s vape approach tendencies can be predicted from brain activity as early as 100‐300 ms after exposure. Findings provide evidence for the presence of fast and automatic processing of vape products, which precedes deliberate processing. Implications for product regulation are provided.

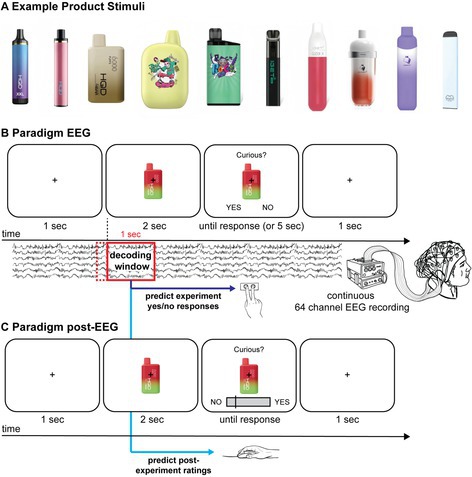

## 1 Introduction

The use of e‐cigarettes (also known as vaping) is increasing globally [1, 2]. These products have been found to contain many harmful substances [3, 4, 5, 6, 7, 8], and a growing body of evidence has linked e‐cigarette use to various short‐ and long‐term harms [9, 10, 11, 12, 13, 14]. A considerable body of evidence also indicates that e‐cigarette use may increase the risk of smoking, with a recent meta‐analysis concluding that people who have never smoked but use e‐cigarettes are approximately three times more likely to initiate tobacco smoking than those who avoid e‐cigarettes [15]. Preventing increases in e‐cigarette use, especially among youth, has thus become a public health priority.

Increases in e‐cigarette use can be partially attributed to product marketing [16, 17], with e‐cigarette manufacturers and distributors using multiple strategies to increase product sales. These tactics span each of the four Ps of marketing: product, place, promotion and price [18]. Of interest to the present study is product‐based marketing. E‐cigarettes are available in thousands of appealing flavours, and product packaging features bright colours, cartoons and playful fonts [19, 20, 21]. Some models feature cartoons and other illustrations on the device itself (e.g., IGET Bars and STIG Cubano), and market scan analyses have identified the availability of device ‘skins’: a customised outer covering or wrap that is applied to the surface of an e‐cigarette to enhance its appearance [21]. Finally, the products are available in different shapes, some of which are designed to resemble stationery items such as USB drives, pens and highlighters.

Despite the likely impact of these product features on product appeal and use intentions, research assessing this impact is limited. Available work suggests that tobacco‐flavoured e‐cigarettes are less likely than other flavours to generate curiosity and willingness to try the product [22, 23, 24], and discrete choice experiments indicate that adolescents who have never used e‐cigarettes are more likely to choose a nontobacco‐flavoured e‐cigarette than a tobacco‐flavoured e‐cigarette [25]. Research exploring product packaging has found that brightly coloured e‐cigarette packaging is considered appealing [26] and contributes to use susceptibility [27].

A key limitation of prior work is a reliance on self‐report measures, responses to which require self‐reflection and insight. Such measures are thus subject to social desirability bias and fail to account for cognitions of which participants are unaware. Cognitive neuroscience methods represent a potential means by which these limitations can be addressed, as they can provide unique and objective insights into the impact of e‐cigarette product features. For example, if e‐cigarette products are perceived as appealing, we would expect these products to elicit a fast neural response that originates in the reward structures of the brain, much like other rewarding primary reinforcers such as food and beverages. We would also expect exposure to trigger approach behaviour. Recent developments in cognitive neuroscience have made it possible to go one step further and use patterns of electrophysiological brain activity recorded at high temporal resolution to predict attitudes toward various objects and products with high precision [28, 29, 30, 31]. These ‘neural decoding’ techniques [32, 33] apply machine learning classifiers to detect regularities in complex brain activity patterns recorded at different points in time. Prior work has used neural decoding to predict approach tendencies such as participants' willingness to consume certain foods and their subjective perceptions of the healthiness and tastiness of these foods [29].

### 1.1 Present Study

Given the promise shown by neural decoding techniques in predicting people's attitudes and intentions in relation to food, we used these techniques to establish whether exposure to e‐cigarette products triggers rapid and automatic product approach tendencies. To achieve this, we conducted an electroencephalography (EEG) study in which we presented young participants with images of e‐cigarette products for several seconds while their brain activity was recorded. We hypothesised that neural activation patterns during the first second of product exposure predict whether participants find the product appealing, are curious about the product and want to use the product. We further hypothesised that the same early neural activation patterns could be used to predict the strength of product appeal, curiosity and wanting in post‐experimental ratings. To explore whether such effects differ based on vaping status, we included in our sample those who currently vape, those who had vaped in the past and those who had never vaped.

## 2 Method

### 2.1 Participants

We recruited 38 young Australians aged 17 to 23 years (mean = 19.05 years, SD = 1.45) from a university in Victoria, Australia. Young people were chosen as the target sample as they are the most prevalent users of e‐cigarettes in Australia [34]. Two‐thirds of the sample (68%) were women. Almost one‐third of the sample (32%) reported having never used e‐cigarettes, 42% reported current use of e‐cigarettes (i.e., use in the last 30 days) and 26% reported former use of e‐cigarettes (i.e., lifetime use but no use in the last 30 days). All participants were right‐handed and had normal or corrected‐to‐normal vision.

### 2.2 Stimuli

**FIGURE 1:**
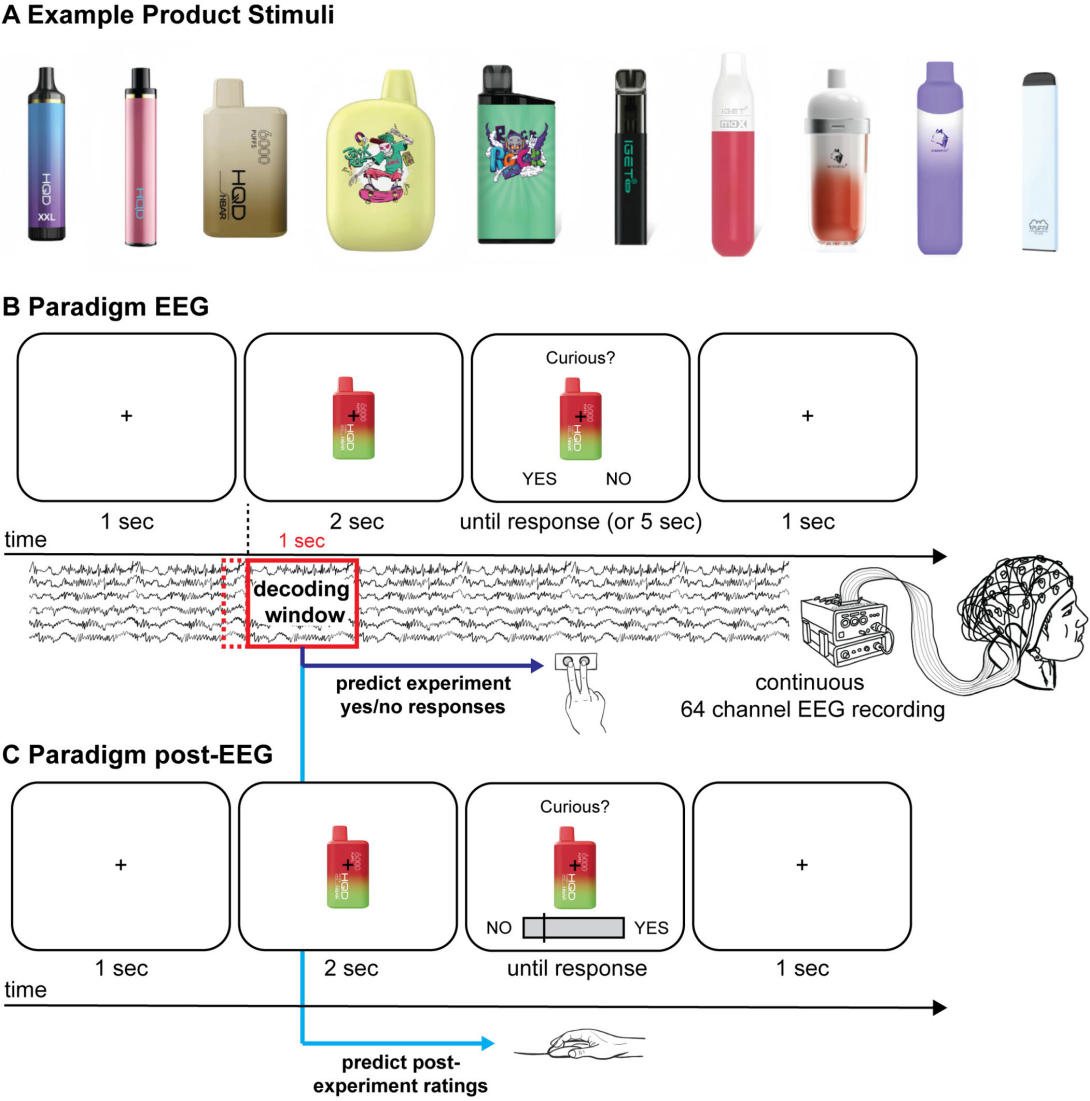
(A) Example stimuli used in the experiment. (B) Experimental paradigm for the EEG experiment. After a 1000‐ms fixation period, participants viewed each product image for 2000 ms before the response options (YES and NO) appeared on the left or right sides, prompting a left or right button press. Participants had a 5000 ms window to make a response. The last 100 ms of data from the fixation period was used as the baseline. EEG data from the first 1000 ms after image presentation was used to train multivariate classifier and regression models to predict Yes/No responses and post‐experimental ratings. After responding, the product image screen was replaced by a fixation screen for 1000 ms before the next trial began. (C) Participants were presented with the same product images in a random order, using the same timing as for the EEG experiment. The only difference was that responses were given using a continuous slider scale, with participants rating the extent to which they found the presented product appealing, were curious about the product and wanted to use the product. The location of the YES and NO ends of the scale was pseudo‐randomised in each trial.

Images of e‐cigarettes were sourced from the websites of various online vape retailers. A total of 120 distinct images were used as stimuli, with the products depicted in the images representing the most widely used e‐cigarettes in Australia (IGET, HQD Cuvie, Gunnpod and Puff Bars). Products varied by device make, flavour and colour. Images were shown in their original colours and displayed on a grey background. No alterations were made to any brand names, device make information or logos that appeared on the device depicted in the image. Flavour descriptors (e.g., ‘cola ice’ and ‘blueberry’) were removed to avoid the participants fixating on this text, which would lead to eye‐movement artefacts in the EEG signal. Images varied slightly in size (depending on the specific products), measuring approximately 600 × 450 pixels. They were presented on a 1680 × 1050 pixel LCD monitor with a screen refresh rate of 60 Hz using Psychtoolbox [35] interfacing MATLAB R2019b. Examples of the images presented to participants can be found in Figure 1A.

### 2.3 Procedure

Participants attended an EEG laboratory located on a university campus. After providing written informed consent, they were seated in front of a computer monitor in a sound‐insulated testing booth with their head location fixed using a chin rest approximately 60 cm from the screen. An EEG cap was then fitted, and participants were provided with detailed instructions about the EEG task to follow. Specifically, they were informed that they would be shown a series of vape products and that for each product they would be asked to respond ‘no’ or ‘yes’ to one of three questions: ‘Is this product appealing?’, ‘Are you curious about using this product?’ and ‘Do you want to use this product?’. They were advised that during the experiment, each question would be prompted by the following indicators: *Appealing? Curious?* and *Want to use?*. They were also informed that the location of the ‘no’ and ‘yes’ response buttons would be randomised to either a left or right button on their computer keyboard.

#### 2.3.1 EEG Protocol

A total of 360 trials were presented across six blocks (60 trials per block), with each block taking approximately 3 min to complete. Each trial began with a 1000‐ms fixation period, after which participants were presented with one of the aforementioned product images for up to 7000 ms (EEG paradigm depicted in Figure 1B). During the first 2000 ms of image exposure, only the product image was displayed on screen. For the remaining 5000 ms, one question probing either appeal (‘appealing?’), curiosity (‘curious?’) or wanting (‘want to use?’) was displayed above the image, while ‘NO’ and ‘YES’ options were displayed below the image on the left or right side. To prevent participants from preparing motor responses during the first 2000 ms of product image exposure, the locations of the NO and YES options were pseudo‐randomised, such that each appeared on the left or right side an equal number of times for each of the three questions across all 120 products. Participants responded by pressing a left (‘N’) or right (‘M’) button on a computer keyboard with their right index or middle finger, respectively, after which the product image disappeared. Their response was followed by a 1000 ms fixation period before the next trial started. Each of the 120 product images was shown in combination with each question in a pseudo‐randomised order, such that the same image did not immediately appear again. Participants completed five practice trials prior to the experiment to familiarise themselves with the nature of the task. Five additional images were used for these practice trials.

#### 2.3.2 Post‐EEG Protocol

After the EEG experiment, the EEG cap was removed, and participants were given the opportunity to wash their hair. They were then seated in front of a computer and the protocol described above was repeated, with two changes: (i) product images were presented in a new, randomised order, and (ii) participants responded to each question using a continuous scale anchored with the responses NO and YES on either side. These anchors were displayed equally often on the left and right side of the scale [as per 29, 36], with participants using a mouse to slide the scale to the position that corresponded with their degree of agreement and confirming their choice with a mouse click (paradigm depicted in Figure 1C). Rating values were not explicitly displayed; instead, responses were recoded offline into scores ranging from 0 (no) to 100 (yes) for subsequent analysis.

After completing the post‐EEG trials, participants were administered a survey that collected information relating to their demographic characteristics (e.g., gender and age) and vaping‐related behaviour (i.e., ever use and use within the past 30 days). They were then debriefed and given their reimbursement (AUD60). The entire experiment took approximately 2.5 h to complete. The study was approved by a university Human Research Ethics Committee (The University of Melbourne: #27971) and conducted in accordance with the Declaration of Helsinki.

#### 2.3.3 EEG Recording and Data Processing

A 64‐channel BioSemi Active II system recorded EEG data at a sampling rate of 512 Hz (recording bandwidth DC‐102 Hz) using common mode sense and driven right leg electrodes (http://www.biosemi.com/faq/cms&drl.htm). To achieve this, Ag/AgCl electrodes were attached to a fabric cap according to the International 10–20 system. Additional electrodes were placed above and below the left eye, 1 cm from the outer canthi of each eye and above the left and right mastoids.

Data preprocessing followed the procedures outlined in Schubert et al. [29] using EEGLab v14.1.2 [37] running in MATLAB. Excessively noisy channels and sections of data containing muscle and skin potential artefacts were identified via visual inspection and removed. Data were referenced to the average of the two mastoids and a high‐pass (0.1 Hz) and low‐pass (30 Hz) filter was applied (EEGLab FIR Filter New, default settings). Independent components analysis [38] was used to identify and remove artefacts associated with eye movements and eyeblinks following guidelines outlined in Chaumon et al. [39]. Excessively noisy channels were removed and interpolated using spherical spline interpolation, with an average of 2.04 channels interpolated. Data were then segmented into epochs beginning 100 ms before an image was presented and ending 1000 ms after image onset. The 100‐ms prestimulus period was used for baseline subtraction. Epochs in which amplitudes at any channel exceeded ±150 μV were excluded from analyses [as per 29].

### 2.4 Analyses

#### 2.4.1 Descriptive

For each of the variables of interest (appeal, curiosity and wanting), descriptive statistics were calculated for the yes/no responses obtained during the EEG experiment and the 0–100 ratings obtained after the EEG experiment. A series of one‐way ANOVAs and Bonferroni‐corrected post hoc tests were conducted to test for differences by participant vaping status.

#### 2.4.2 Multivariate Decoding Analyses

The multivariate analyses closely followed procedures outlined in Schubert et al. [29] and have been described in detail in more technical papers [32, 40]. We used the Decision Decoding Toolbox v1.0.5 [32] to conduct two types of analyses. For the first analysis, support vector machine (SVM) classification analyses were conducted on single‐trial event‐related potential (ERP) data extracted from the first second following product image presentation. This was applied to predict the Yes vs. No responses provided by participants during the EEG experiment. For the second analysis, support vector regression (SVR) analyses were conducted on the same EEG data to predict the 0–100 ratings provided by participants after the EEG experiment. Given there were three questions (appealing, curiosity and wanting) asked of participants, three independent SVM classification analyses and three independent SVR analyses were conducted.

##### 2.4.2.1 SVM Classification

Analyses were conducted for each participant separately. We used a moving‐window approach in which the epoched single‐trial ERP data was analysed using multiple analysis time windows of 10 ms duration covering the first 1000 ms of product presentation, plus the 100 ms baseline period preceding presentation. A linear SVM model (interfacing LIBSVM [41]) was trained on a randomly selected 90% subset of the data, then tested on the remaining 10%. This process was repeated using different 10% subsets for testing the classifier (tenfold cross‐validation). This cross‐validation procedure was then repeated 10 times with EEG epochs randomly assigned to each fold. The average classification accuracy served as a measure for how well the respective time window of ERP data could predict the Yes/No responses. Note that the average classification accuracy is not an absolute measure for how much information is contained in neural patterns, as the model is not optimised to achieve high predictive accuracy (after seeing these results) to avoid the risk of overfitting or producing artificially high predictive accuracy. Instead, this approach is based on establishing the general presence of predictive information by statistical significance testing [32]. For this, an empirical null distribution for statistical testing was obtained by repeating these analyses with randomly shuffled assignment of condition labels to epochs of EEG data. Group‐level statistical tests were conducted using one‐tailed paired‐samples *t*‐tests comparing original and permuted‐labels classification accuracy. Cluster‐based permutation tests were used to correct for multiple comparisons across time windows (see Supporting Information for a more detailed description of the SVM approach). This approach was identical for each of the three SVM classification analyses. In addition, we analysed the feature weights, which indicate the relative importance of different channels (features) to the overall prediction in significant time windows [32]. We focussed on the first significant clusters for each dimension (see Supporting Information for a more detailed description of the analysis). Note that this analysis cannot reveal which brain structures are involved in the process, but it can provide some insights for future neuroimaging studies.

##### 2.4.2.2 SVR Analyses

The SVR analyses were conducted separately for each participant. The overall approach was similar to the SVM classification described above. The only difference was that a linear SVR model (interfacing LIBSVM [41]) was trained to predict the post‐experimental product ratings participants provided after the EEG experiment. For each analysis time window, the SVR outputs a Fisher‐Z transformed correlation coefficient indexing the correlation between the real ‘labels’ (e.g., the appeal ratings) and the predicted ‘labels’ (e.g., the model predicted appeal ratings). For the SVM group‐level analyses, one‐tailed paired‐samples *t*‐tests were used to compare correlation coefficient values across the original and permuted‐labels analyses (see Supporting Information for more details).

## 3 Results

### 3.1 Descriptive

Descriptive statistics for participants' (i) yes and no responses during the EEG experiment and (ii) 0–100 ratings after the EEG experiment are presented in Table 1. Results are presented for the overall sample and stratified by vaping status. One‐way ANOVAs followed by Bonferroni‐corrected post hoc tests were performed to explore differences in responses by vaping status. Results (summarised in Table 1, with full results presented in Table S1) indicated that those who currently use vapes provided significantly more ‘Yes’ responses, significantly fewer ‘No’ responses and significantly higher ratings across all three dimensions relative to those who had never vaped. Those who currently use vapes also provided significantly more ‘Yes’ responses, significantly fewer ‘No’ responses and significantly higher ratings on the dimensions of curiosity and wanting relative to those who had vaped in the past. There were no statistically significant differences between those who vaped in the past and those who had never vaped.

**TABLE 1:** Descriptive statistics for choices (Yes/No) and ratings (0–100), stratified by vaping status (summary of significance test results provided).

	Appealing (*N* trials per participant = 360)						
	Mdn	Min	Max	Mdn	Min	Max	Rating M (SD)
	Yes	Yes	Yes	No	No	No	
Full sample (*N* = 38)	95	0	321	249	30	359	29.14 (23.93)
Never vaped (*n* = 12)	61^a^	0	219	282^a^	171	359	9.98^a^ (10.48)
Currently vaping (*n* = 16)	140	6	321	212	30	354	48.12 (22.63)
Past vaping (*n* = 10)	79	0	163	268	192	353	21.78^a^ (13.26)
Past/never combined (*n* = 22)	77	0	219	279	171	359	15.34 (13.00)

	Curiosity (*N* trials per participant = 360)						
	Mdn	Min	Max	Mdn	Min	Max	Rating M (SD)
	Yes	Yes	Yes	No	No	No	
Full sample (*N* = 38)	95	0	315	256	36	357	27.49 (25.19)
Never vaped (*n* = 12)	39^a^	0	200	302^a^	158	357	7.27^a^ (10.10)
Currently vaping (*n* = 16)	149^b^	6	315	199^b^	36	354	47.83 (23.64)
Past vaping (*n* = 10)	66	3	159	278	201	354	19.21^a^ (14.04)
Past/never combined (*n* = 22)	63	0	200	288	158	357	12.70 (13.22)

	Wanting (*N* trials per participant = 360)						
	Mdn	Min	Max	Mdn	Min	Max	Rating M (SD)
	Yes	Yes	Yes	No	No	No	
Full sample (*N* = 38)	56	0	312	283	33	359	24.11 (25.05)
Never vaped (*n* = 12)	20^a^	0	162	323^a^	194	359	5.01^a^ (7.73)
Currently vaping (*n* = 16)	120^b^	6	312	225^b^	33	354	44.83 (24.19)
Past vaping (*n* = 10)	39	0	153	311	207	349	13.87^a^ (12.89)
Past/never combined (*n* = 22)	26	0	162	325	194	359	9.04 (11.08)

Abbreviations: M = mean; Max = maximum; Mdn = median; Min = minimum; SD = standard deviation.

^a^ Significantly different to those who currently vape.

^b^ Significantly different to those who vaped in the past.

A series of Pearson correlations was used to confirm that the proportion of Yes/No responses during the EEG experiment across the entire sample were indeed correlated with the average post‐experiment ratings for the same images for each question. Results are presented in the online Supporting Information. All ratings (i.e., appealing, curiosity and wanting) were significantly and positively correlated with the number of Yes responses and negatively correlated with the number of No responses.

### 3.2 EEG Decoding Analyses

#### 3.2.1 SVM Classification

**FIGURE 2:**
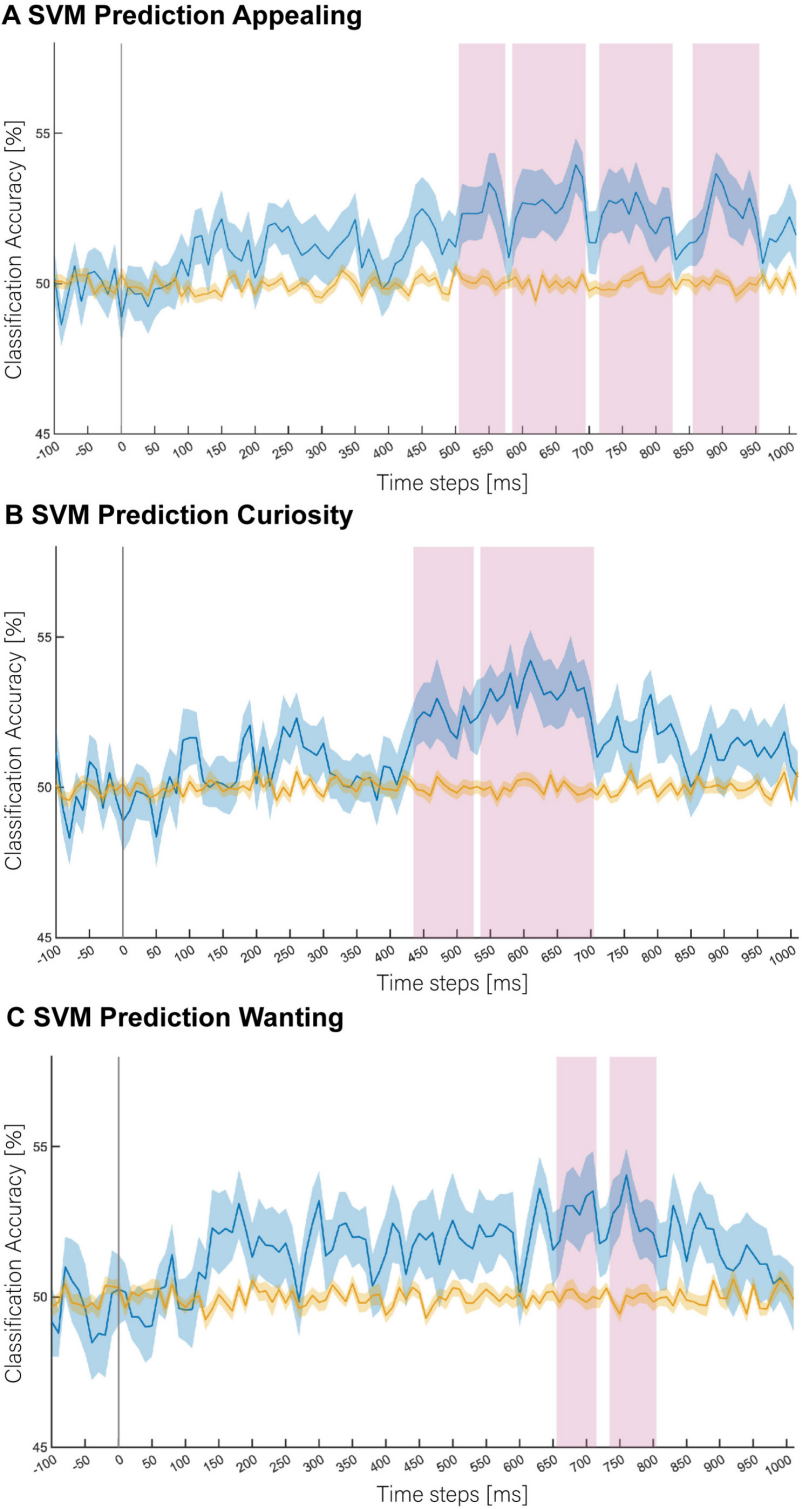
SVM classification results for the dimensions of (A) *Appealing*, (B) *Curiosity* and (C) *Wanting*. Time point 0 ms corresponds to the onset of image presentation. Blue lines represent classification accuracy (with shaded regions showing S.E.M.) between predicted and true labels (chance level is 50%). Yellow lines represent decoding results based on the same analyses using shuffled labels (i.e., the empirical chance distribution) that were used for statistical testing. Pink vertical bars highlight time points at which the decoding results significantly differed from the shuffled decoding results (*p* < 0.05, corrected for multiple comparisons) and classifiers successfully predicted participants' Yes/No decisions based on the EEG data. Data of participants that responded ‘Yes’ to less than 20 images were removed as trial numbers were too low to train the classifier for both classes. Of the full sample, *n* = 33 were included in the decoding analysis of *appealing*, *n* = 31 were included in the decoding analysis of *curiosity* and *n* = 26 were included in the decoding analysis of *wanting*.

Results from the SVM classification analyses conducted on the yes/no responses for each of the dimensions of *appealing*, *curiosity* and *wanting* are presented in Figure 2. For all three dimensions, classification accuracy was above chance within the first second of image presentation, even after correcting for multiple comparisons across analysis time windows. Similar prediction time courses were found for all dimensions, with above‐chance prediction between 440 and 860 ms from product image presentation. The increase in prediction accuracy was observed from 100 to 150 ms for all three dimensions, albeit only when multiple comparisons corrections were not applied. Feature weight analyses were conducted for the first significant clusters (after corrections for multiple comparisons) emerging for each dimension (*appealing* 520–580 ms; *curiosity* 450–530 ms; *wanting* 670–720 ms). These analyses revealed significant feature weights at frontal channels as well as occipital and lateral parietal channels (Figure S1).

#### 3.2.2 SVR

**FIGURE 3:**
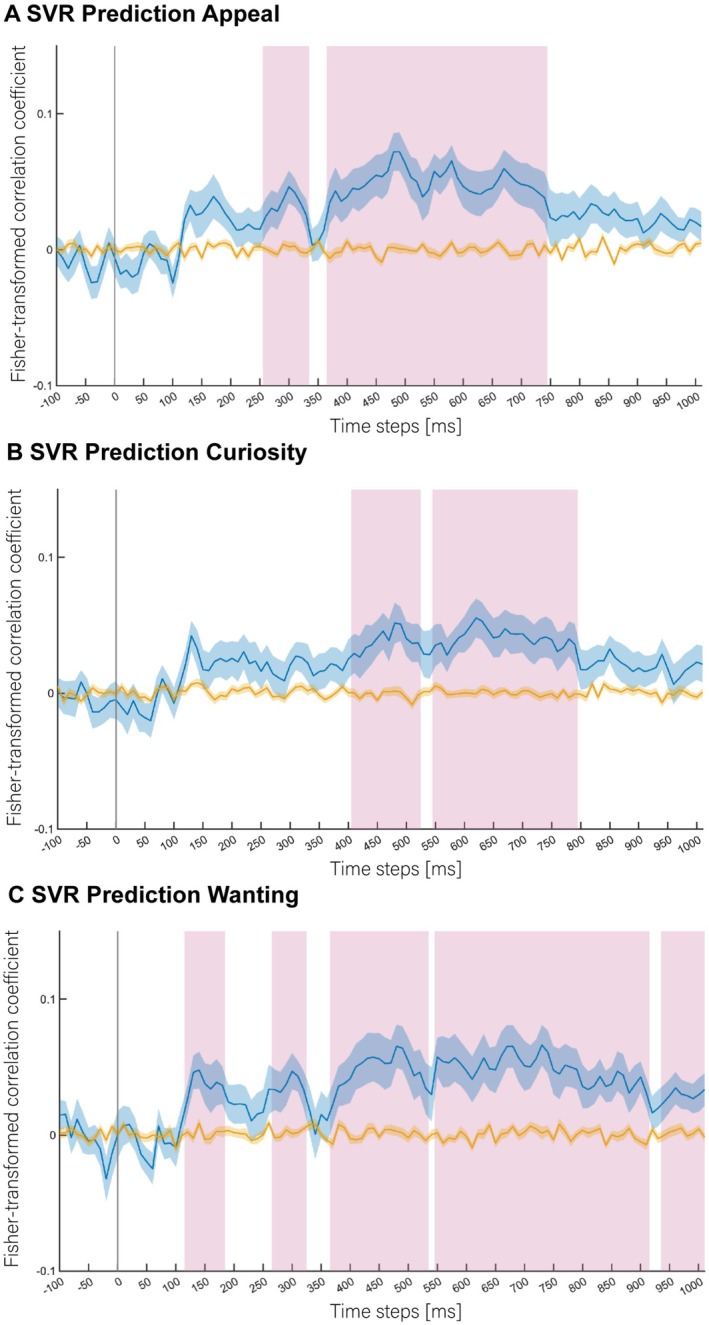
SVR analysis results for the dimensions (A) *Appealing*, (B) *Curiosity* and (C) *Wanting*. Time point 0 ms corresponds to image presentation onset. Blue lines represent Fisher‐transformed correlation coefficients (with shaded regions showing S.E.M.) for the correlation between predicted and true labels (chance level is *Z* = 0). Yellow lines represent decoding results based on the same analyses using shuffled labels (i.e., the empirical chance distribution) that were used for statistical testing. Pink vertical bars highlight time points at which the decoding results significantly differed from the shuffled decoding results (*p* < 0.05, corrected for multiple comparisons) denoting above‐chance prediction of the post‐experimental ratings based on the EEG data. Data of participants that did not produce sufficiently variable post‐experimental ratings for the respective dimensions to fit the regression model were removed. Of the full sample, *n* = 32 were included in the decoding analysis of *appealing*, *n* = 31 were included in for decoding analysis of *curiosity* and *n* = 25 were included in for decoding analysis of *wanting*.

Results from the SVR analyses conducted on the 0–100 ratings for each of the dimensions of *appealing*, *curiosity* and *wanting* are presented in Figure 3. Overall, the SVR results supported the classification results, with clusters of above‐chance prediction occurring across time windows between 370 and 1000 ms relative to product image presentation for all three dimensions. Appeal and wanting could also be predicted using EEG signals in clusters of time windows between 110 and 320 ms (Figure 3).

**FIGURE 4:**
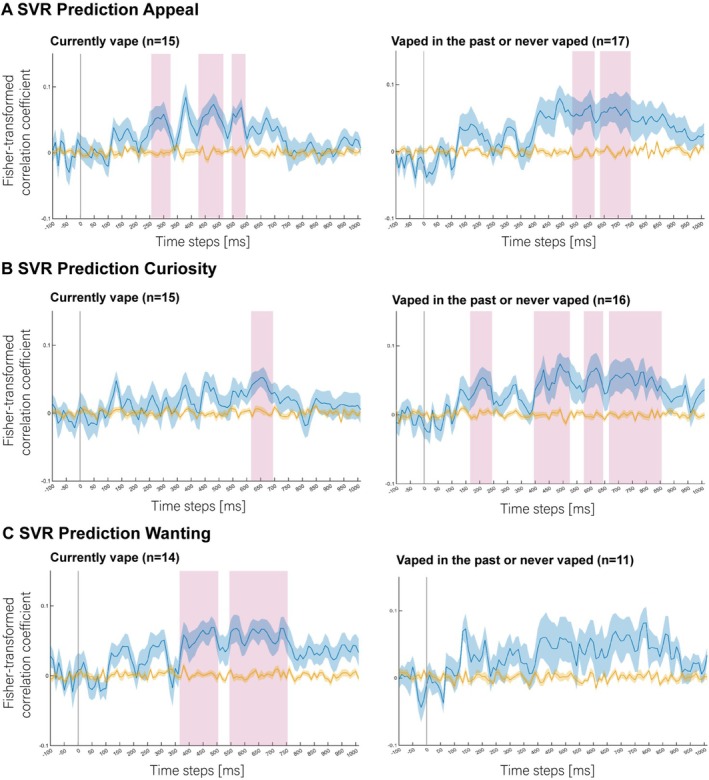
SVR analysis results for the dimensions (A) *Appealing*, (B) *Curiosity* and (C) *Wanting* for participants who currently vape (left panels) and participants who vaped in the past or never vaped (right panels). Time point 0 ms corresponds to image presentation onset. Blue lines represent Fisher‐transformed correlation coefficients (with shaded regions showing S.E.M.) for correlations between predicted and true labels (chance level is *Z* = 0). Yellow lines represent decoding results based on the same analyses using shuffled labels (i.e., the empirical chance distribution) that were used for statistical testing. Pink vertical bars highlight time points at which the decoding results significantly differed from the shuffled labels decoding results (*p* < 0.05, corrected for multiple comparisons) denoting above‐chance prediction of the post‐experimental ratings based on the EEG data. Sample sizes depend on sufficiently variable post‐experimental ratings for the respective dimensions to fit the regression model for each group.

Sensitivity analyses were conducted to determine whether the results varied by vaping status. Given the high similarity between results from both multivariate analyses, we focussed only on SVR results. We first split the sample into two groups: (i) those who currently vape and (ii) those who had never vaped or had vaped in the past. We then repeated the SVR analyses on each group. Note that due to the small sample sizes at the subgroup level, the following results should be interpreted with caution. For the SVR analysis of *appealing* ratings, above‐chance prediction was observed in both groups (Figure 4A). For the SVR analysis of *curiosity* ratings, above‐chance decoding was observed for both groups; however, a larger range of above‐chance decoding time windows was observed for those who had never vaped or had vaped in the past. Finally, for the SVR analysis of *wanting* ratings, above‐chance decoding was observed for those who currently vape, while the decoding performance for those who never vaped or vaped in the past was not significantly above‐chance after correction for multiple comparisons.

## 4 Discussion

We sought to determine whether exposure to images of vape products evokes patterns of brain activity in young people that are indicative of strong approach motivation. To achieve this aim, we used multivariate analysis of EEG signals recorded during the first second of exposure to predict whether participants find a given vape product appealing, are curious about the product and want to use the product. We also tested whether brain activity from the same time windows predicts more fine‐grained post‐experimental ratings of vape products.

Results from both SVM and SVR analyses suggest that young people's vape approach tendencies can be predicted from brain activity as early as 100–300 ms after exposure, and then again over a sustained time window spanning 350–800 ms. These findings were not restricted to those who currently vape, with similar patterns observed in young people who had never vaped or had tried vape products in the past. More specifically, neural data from those who currently vape, as well as those who never vaped or had vaped in the past, could be used to predict product appeal and curiosity. Ratings for product wanting were best predicted in those who currently vape.

These findings provide neuroscientific evidence for the presence of fast, automatic processing of vape products, which results in immediate approach tendencies. This indicates that approach attitudes and behavioural intentions that occur in response to exposure to vape products precede slower forms of deliberate reasoning processes, consistent with prior work conducted on palatable food products [29, 42, 43, 44]. Importantly, our findings go beyond simply showing that vape products draw attention: they provide direct evidence that, within the first second of cognitive processing, neural signals already reflect different aspects of approach motivation. The demonstration of such a fast semantic analysis of consumer products is a major advancement on previous work exploring responses to food products and, to our knowledge, is the first demonstration of this effect for vape products. In addition to demonstrating that we can predict whether participants are drawn to vape products, we showed that we can predict how appealing a potential consumer finds a vape product, how much they want to consume it and how curious they are about the product. Our results further indicate that these core aspects of opinion formation and decision‐making are processed before consumers can potentially engage the deliberate cognitive processes required to counteract the effects of vape product design, providing key evidence that vape product designs are highly successful in triggering immediate approach responses. This finding is of high relevance to the cognitive neuroscience of addiction, as it shows that multivariate pattern analysis of EEG data has the potential to be optimised for predicting the effects of addictive products on individuals.

Our results have key implications for vaping prevention initiatives. First, our findings support the elimination of product‐based e‐cigarette marketing and the introduction of standardised or plain packaging that extends to the device itself, not just the box in which the device is sold. Second, results support the introduction of regulations that limit young people's exposure to e‐cigarettes and related advertising. Such action includes restrictions on advertising and ensuring e‐cigarettes are not readily available, especially from stores that are typically frequented by young people. Ensuring that products are not visible to passers‐by and are instead hidden from view is another consideration. Such an approach is consistent with tobacco display bans, a policy measure that has been found to reduce (i) impulse purchases, (ii) smoking susceptibility and (iii) smoking‐related behaviours [45, 46, 47, 48]. In countries that have already implemented product‐based regulations and/or introduced advertising restrictions and display bans for e‐cigarette products (e.g., Denmark, Finland and Australia), our findings highlight the importance of enforcement to ensure policy effectiveness.

### 4.1 Limitations and Strengths

Some key limitations should be considered when interpreting the findings. First, while the sample size was sufficient for the analyses conducted, it was too small to explore whether any additional factors such as smoking status or gender influence the processing of vape products. An examination of this with a larger sample is recommended. Second, the age range of participants was narrow, precluding analyses based on this variable. Future research could seek to include a broader sample and investigate the presence of any differences in the onset of neural approach responses between different age groups. Third, we omitted classical ERP analysis in this study because our focus was on directly predicting the outcome of the fast semantic product evaluation from brain signals. While this was the appropriate and more sensitive approach for our research question, future studies could also use a modified paradigm optimised for classical ERP analysis to investigate differences in early sensory and attentional processing. Fourth, we did not investigate where in the brain predictive signals originate. While the feature weight analysis shows the importance of frontal, occipital and lateral parietal channels for the prediction, these results cannot be interpreted as detecting informative underlying structures. It is important to note that our approach was not designed to localise predictive brain systems, which requires techniques with higher spatial resolution, such as magnetoencephalography (MEG). Finally, we acknowledge that all well‐marketed products trigger approach tendencies, and that it should be possible to decode their effects from neural data [28, 29, 31]. However, as noted, the explicit demonstration of this effect for vape products is of critical importance to policymakers who require such evidence in their decision‐making deliberations.

A key strength of this study is the methodological approach adopted. Instead of analysing classical neural indices of approach motivation, which can be ambiguous in their meaning, we used multivariate analysis approaches to directly predict whether—and how strongly—participants find the vape products to which they were exposed appealing, are curious about the products and want to consume the products. Importantly, our analysis focussed on data recorded during the first second of product exposure, before participants were asked a specific question about the products. This allowed us to assess whether desire for the products is automatically and rapidly triggered. Such an approach could be used to assess whether interventions such as health warning messages assist with modifying processing and change neural representations and early stages of decision‐making, as has been demonstrated in the area of food [44]. This would be a valuable addition to the literature and has the potential to provide policymakers with key information on the effectiveness of warning messages.

### 4.2 Conclusion

We used innovative cognitive neuroscience methods to investigate approach motivation among young people exposed to images of e‐cigarette products. The finding that brain activity patterns predicted various and detailed aspects of approach motivation early after product exposure provides strong evidence that exposure to e‐cigarette products has an immediate effect on young people's neural processing. This novel neural approach to understanding the impact of e‐cigarette marketing offers valuable insights into how fast and automatic the processing of product‐based marketing of e‐cigarettes unfolds in young people's brains.

## Author Contributions

S.B. acquired funding, conceptualised and designed the study, conducted the analyses, and co‐wrote the manuscript. D.F. supervised data acquisition and preprocessing, and reviewed and edited the manuscript. J.Y. collected data, pre‐processed the data, and reviewed and edited the manuscript. M.I.J. acquired funding, conceptualised the study, and co‐wrote the manuscript. All authors have read and approved the final manuscript.

## Funding

This work was funded by the Melbourne School of Psychological Sciences. The funding source was not involved in the study design; in the collection, analysis and interpretation of data; in the writing of the article; and in the decision to submit the article for publication. Authors D.F. and M.J. are currently employed by the Melbourne School of Psychological Sciences. Author S.B. was employed by the Melbourne School of Psychological Sciences at the time funding was received. M.J. is supported by a National Health and Medical Research Council Investigator Grant (APP1194713). D.F. is supported by an Australian Research Council Discovery Early Career Researcher Award (ARC DE220101508).

## Ethics Statement

The study was approved by a university Human Research Ethics Committee (The University of Melbourne: #27971) and conducted in accordance with the Declaration of Helsinki.

## Consent

The authors have nothing to report.

## Conflicts of Interest

The authors declare no conflicts of interest.

## Supporting information




**Table S1:** ANOVA results for differences in choice proportions (Yes/No) and ratings (0–100) by vaping status.
**Figure S1:** Feature weight analysis results for the first significant SVM classification clusters for the dimensions of (A) Appealing (520–580 ms), (B) Curiosity (450–530 ms) and (C) Wanting (670–720 ms). The top row displays the thresholded topographical significance maps, with significant absolute standardised features highlighted in light blue (*p* < 0.05). The bottom row shows detailed feature weight matrices for the single analysis time windows contained in the same respective clusters.

## Data Availability

Data will be made available upon reasonable request. Data will not be shared with the tobacco or vape industries and their affiliates.
