# Supplementary material for: Exposure to Vape Products Elicits Neural Activity Patterns Indicative of Approach Motivation Among Young People
Source: Addict Biol. 2026 Feb 16;31(2):e70130. doi: 10.1111/adb.70130 (PMC12909270; doi:10.1111/adb.70130)
Supplement: Supplementary file 1 — Table S1: ANOVA results for differences in choice proportions (Yes/No) and ratings (0–100) by vaping status. Figure S1: Feature weight analysis results for the first significant SVM classification clusters for the dimensions of (A) Appealing (520–580 ms), (B) Curiosity (450–530 ms) and (C) Wanting (670–720 ms). The top row displays the thresholded topographical significance maps, with significant absolute standardised features highlighted in light blue (p < 0.05). The bottom row shows detailed feature weight matrices for the single analysis time windows contained in the same respective clusters. [file ADB-31-e70130-s001.pdf]

SUPPLEMENTARY MATERIALS

EXPOSURE TO VAPE PRODUCTS ELICITS NEURAL ACTIVITY PATTERNS INDICATIVE OF APPROACH MOTIVATION AMONG YOUNG PEOPLE

Stefan Bode[a], Daniel Feuerriegel[b], Jane Yook[b], Michelle Jongenelis[c]*

[a] Psychology, Division of Science
New York University Abu Dhabi, Saadiyat Campus
Saadiyat Marina District, Abu Dhabi, United Arab Emirates
sb10382@nyu.edu

[b] Melbourne School of Psychological Sciences,
The University of Melbourne,
Grattan Street, Parkville, Victoria 3010
Australia
daniel.feuerriegel@unimelb.edu.au

[b] Melbourne School of Psychological Sciences,
The University of Melbourne,
Grattan Street, Parkville, Victoria 3010
Australia
jane.yook@unimelb.edu.au

[c] Melbourne Centre for Behaviour Change, Melbourne School of Psychological Sciences
The University of Melbourne,
Grattan Street, Parkville, Victoria 3010
Australia
michelle.jongenelis@unimelb.edu.au

*CORRESPONDING AUTHOR

SUPPLEMENTARY METHODS

SVM classification

The approach used for SVM classification was identical for each of the three SVM classification analyses. Analyses were conducted for each participant separately. We used a moving-window approach in which the epoched single-trial ERP data was analysed within an analysis time window of 10ms width, which was moved through the epoch in 10ms non-overlapping steps. Within each analysis window, the five data points were averaged for each 64 EEG channel ('spatial analysis' setting in the toolbox) and transformed into a vector that represented the specific spatial brain activity pattern (with 64 features) for this time window. All vectors from the same analysis time window were then sorted into two classes according to the response (Yes vs. No) participants gave for the respective product that was shown in any given trial. Considering that responses were not balanced (e.g., the participant might have responded "Yes" less often), the toolbox randomly selected the same number of trials for the class with higher numbers (in this example, "No" responses) to match the class with lower numbers, meaning that patterns representing each class were equally often represented in the full set. A linear SVM model (standard cost parameter C = 1, interfacing LIMSVM [1]) was trained on a randomly selected 90% of the data, then tested on the remaining 10%. This process was repeated independently using a 10-fold cross-validation procedure until all 10% data subsets had been used as test data once while training on all other subsets (importantly, each time a new model was trained and tested). The cross-validation process itself was then repeated ten times with newly drawn random data to obtain a conservative estimate of the prediction based on average classifier performance from all 10×10 iterations, circumventing any potential drawing biases. The final classification accuracy that served as a measure for how well the respective time window of ERP data could predict the Yes/No responses was therefore the average of 100 independent analyses.

Next, the analysis time window was shifted by 10ms (as described above), and the entire classification procedure was repeated. After conducting this procedure for all 110 analysis time windows in the epoch (i.e. the first 1,000ms of product presentation, plus the 100ms baseline period preceding the image), this resulted in an information time course displaying the classification accuracy over time. Accuracy values above 50% indicate that there was information in the neural activity patterns that allowed prediction of subsequent Yes/No decisions (as chance level was 50%). An empirical test distribution for statistical testing was

obtained by repeating all iterations of the same cross-validation procedure, with exactly the same data and the same labels (i.e., Yes/No responses), the only difference being that the assignment of labels to data was randomised (and freshly randomised for each iteration of the cross-validation procedure). This approach is more conservative than testing against the theoretical chance level [2] and it also controls for any biases inherent in the data [3]. After running these analyses for all participants, group-level statistical tests were conducted using one-tailed paired-samples *t*-tests that compared classification accuracy across the original and shuffled-labels analyses. We corrected for multiple comparisons (i.e., the 110 tests needed to cover the 1,100ms in 10ms time-steps) using cluster-based permutation tests based on the cluster mass statistic, which takes advantage of the statistical non-independence of the adjacent analysis time windows (see [3, 4], cluster inclusion alpha = .05, 5,000 permutation samples drawn to estimate the cluster-level null distribution).

Finally, we conducted feature weight analyses [3] for the first significant cluster for each of the three dimensions/SMV analyses, respectively. These consisted of: *Appealing* 520-580 ms; *curiosity* 450-530 ms; and *wanting* 670-720 ms. Feature weights describe the contribution of each feature (here: channels) in determining the decision boundary for the classification (separating the classes, i.e. products associated with Yes/No responses). Since the raw feature weights derived from SVMs are prone to erroneous interpretations, they were corrected by employing the algorithm described by Haufe et al. (2014) [5]. Since we used spatial decoding analyses, each of the 64 head channels was a feature and was assigned a weight for each analysis time window. We then standardised the absolute feature weights for each analysis time window to make them comparable, and we averaged over each channel's weights across the analysis time windows that were included in the significant cluster. As a last step, we tested all standardised feature weights against zero using one-sided t-tests ($p < .05$) to determine which channels significantly contributed to the prediction. The threshold maps and a detailed feature weight matrix for the respective clusters are presented in Supplementary Figure S1. Note, however, that this analysis ultimately cannot reveal which underlying brain structures are involved in the cognitive processes that produce the predictive brain signals.

SVR analyses

The SVR analyses were conducted separately for each participant. The overall approach was similar to the SVM classification described above. The ERP data used was identical and covered the 100ms baseline pre-image period and the first 1,000ms of product image presentation. The

same moving-window approach was applied, again using a 10ms wide analysis time window that was moved in non-overlapping steps through the epoched data. The same ten-fold cross-validation procedure with ten iterations was used for each time step. The only difference was that a linear SVR model (standard cost parameter C = 0.1, interfacing LIMSVM [1]) was trained to predict the post-experimental ratings the participants provided after the EEG experiment for the respective products. In other words, for a given analysis (e.g., appealing), ERP data from the first second of image presentation was used (in steps of 10ms) to predict how the participant rated the products on this dimension. For each analysis time window, the SVR outputs a Fisher-transformed correlation coefficient for the correlation between the real "labels" (i.e., the appealing rating) and the predicted "label" (i.e., the predicted appealing rating). The average result of all iterations of all cross-validation steps was the final output and assigned to the respective analysis time window. As before, an identical analysis was then repeated for the data from each analysis time window until the end of the epoch. The same procedure (including all ten iterations of the ten-fold cross-validation procedure) was then repeated for all analysis time windows using the same data and the same labels, but a shuffled assignment of labels to data, to obtain an empirical chance distribution of random results or statistical testing. As for the SVM, the final step was conducting group-level statistical tests using one-tailed paired-samples *t*-tests comparing correlation coefficients across the original and shuffled labels analyses. We corrected for multiple comparisons using cluster-based permutation tests based on the cluster mass statistic [3, 4].

SUPPLEMENTARY RESULTS

ANOVA results for analyses assessing differences by vaping status

Table S1: *ANOVA results for differences in choice proportions (Yes/No) and ratings (0-100) by vaping status*

	ANOVA		Post-hoc tests (*p* [CI% 95])		
**Outcome variable**	**F(2,35)**	***p***	**current vaping cf. never vaped**	**current vaping cf. past vaping**	**past vaping cf. never vaped**
% Yes Appealing	5.52	**.008**	**.014** [13.52, 147.94]	.053 [-0.68, 141.21]	1.00 [-64.89, 85.82]
% No Appealing	6.22	**.005**	**.006** [-156.18, -21.53]	.060 [-139.91, 2.23]	1.00 [-95.51, 55.47]
% Yes Curiosity	7.39	**.002**	**.004** [27.74, 167.47]	**.022** [9.89, 157.38]	1.00 [-64.36, 92.30]
% No Curiosity	7.02	**.003**	**.004** [-168.00, 26.63]	**.031** [-155.23, -6.00]	1.00 [-95.96, 62.56]
% Yes Wanting	8.12	**.001**	**.002** [36.09, 188.29]	**.017** [13.90, 174.57]	1.00 [-67.38, 103.28]
% No Wanting	9.55	**<.001**	**<.001** [-194.83, -45.71]	**.010** [-176.85, -19.43]	1.00 [-105.73, 61.47]
Rating Appealing	17.90	**<.001**	**<.001** [21.53, 54.75]	**.002** [8.81, 43.87]	.360 [-6.82, 30.42]
Rating Curiosity	18.94	**<.001**	**<.001** [23.32, 57.79]	**.001** [10.42, 46.81]	.388 [-7.39, 31.26]
Rating Wanting	19.69	**<.001**	**<.001** [22.85, 56.79]	**<.001** [13.05, 48.87]	.784 [-10.16, 27.89]

Correlations between Yes/No responses and ratings

A series of Pearson correlations were used to confirm that the proportion of Yes/No responses during the EEG experiment were indeed correlated with the average post-experiment ratings for the respective dimensions. In other words, we analysed (i) whether items that received a higher proportion of Yes responses during the EEG part of the experiment across the entire sample received higher ratings after the experiment and (ii) whether items that received a higher proportion of No responses during the EEG part of the experiment received lower ratings after the experiment. *Appealing* ratings were significantly and positively correlated with the proportion of Yes responses for a given e-cigarette product (r = .79, p < .001) and significantly and negatively correlated with the proportion of No responses (r = -.81, p < .001). *Curiosity* ratings were significantly positively correlated with the proportion of Yes responses (r = .84, p < .001) and significantly negatively correlated with the proportion of No responses (r = -.84, p < .001). Finally, *wanting* ratings were significantly positively correlated with the proportion of Yes responses (r = .84, p < .001) and significantly negatively correlated with the proportion of No responses (r = -.85, p < .001).

Correlations between rating dimensions

Correlation analyses were conducted to confirm that the three dimensions of *appealing*, *curiosity*, and *wanting* were highly related aspects of product approach attitudes and

behavioural intentions. Ratings of product appeal were strongly and positively correlated with ratings of product curiosity ($r = .97, p < .001$) and wanting ($r = .97, p < .001$). Ratings of product curiosity were strongly and positively correlated with ratings of product wanting ($r = .94, p < .001$).

Feature weights analysis

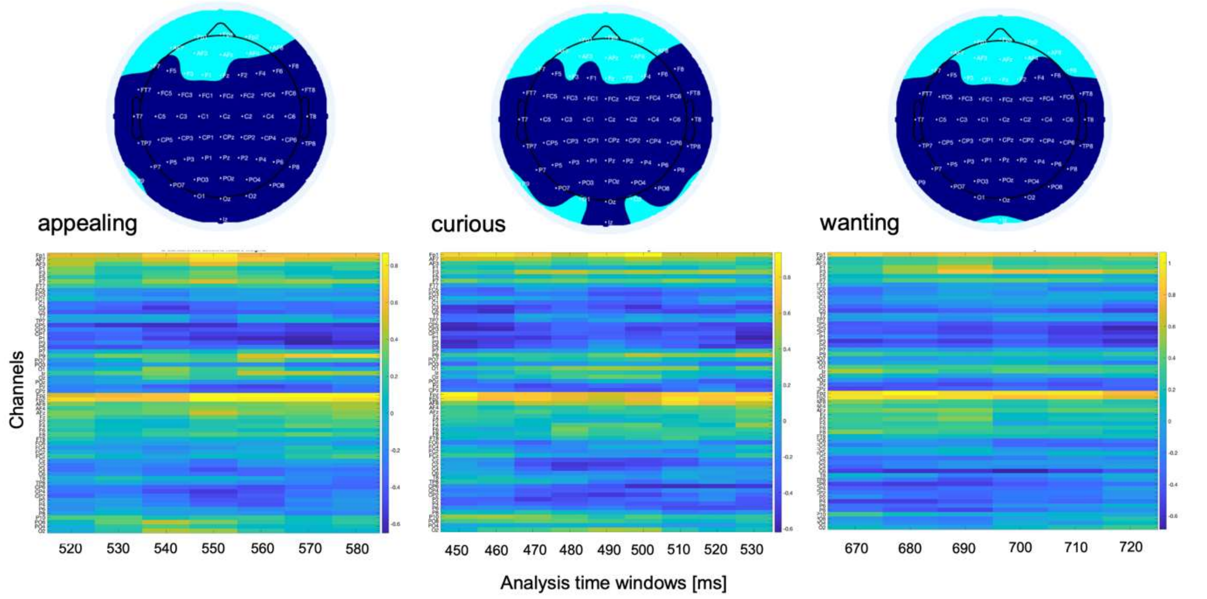

Supplementary Figure S1: Feature weight analysis results for the first significant SVM Classification clusters for the dimensions of A) *Appealing* (520-580ms), B) *Curiosity* (450-530ms), and C) *Wanting* (670-720ms). The top row displays the thresholded topographical significance maps, with significant absolute standardised features highlighted in light blue ($p < .05$). The bottom row shows detailed feature weight matrices for the single analysis time windows contained in the same respective clusters.

SUPPLEMENTARY REFERENCES

1. Chang CC, Lin CJ. LIBSVM: a library for support vector machines. *ACM Trans on Intell Syst Technol*. 2011; 2(3):1-27. doi: 10.1145/1961189.1961199.
2. Combrisson E, Jerbi K. Exceeding chance level by chance: The caveat of theoretical chance levels in brain signal classification and statistical assessment of decoding accuracy. *J Neurosci Methods*. 2015; 250:126-36. doi: 10.1016/j.jneumeth.2015.01.010.
3. Bode S, Feuerriegel D, Bennett D, Alday PM. The Decision Decoding ToolBOX (DDTBOX)–A multivariate pattern analysis toolbox for event-related potentials. *Neuroinformatics*. 2019; 17(1):27-42. doi: 10.1007/s12021-018-9375-z.
4. Bode S, Schubert E, Hogendoorn H, Feuerriegel D. Decoding continuous variables from event-related potential (ERP) data with linear support vector regression using the Decision Decoding Toolbox (DDTBOX). *Front Neurosci*. 2022; 16. doi: 10.3389/fnins.2022.989589.
5. Haufe S, Meinecke F, Görgen K, Dähne S, Haynes JD, Blankertz B, Bießmann F. On the interpretation of weight vectors of linear models in multivariate neuroimaging. *Neuroimage*. 2014; 87:96-110. doi: 10.1016/j.neuroimage.2013.10.067.
